# Differences in Gut Virome Related to Barrett Esophagus and Esophageal Adenocarcinoma

**DOI:** 10.3390/microorganisms9081701

**Published:** 2021-08-10

**Authors:** Tianli Ma, Jinlong Ru, Jinling Xue, Sarah Schulz, Mohammadali Khan Mirzaei, Klaus-Peter Janssen, Michael Quante, Li Deng

**Affiliations:** 1 Helmholtz Centre Munich—German Research Center for Environmental Health, Institute of Virology, 85764 Neuherberg, Germany; tianli.ma@helmholtz-muenchen.de (T.M.); jinlong.ru@helmholtz-muenchen.de (J.R.); jinling.xue@helmholtz-muenchen.de (J.X.); sarah.schulz@helmholtz-muenchen.de (S.S.); m.khanmirzaei@helmholtz-muenchen.de (M.K.M.); 2 Institute of Virology, Technical University of Munich, 81675 Munich, Germany; 3 Department of Surgery, Klinikum Rechts der Isar, Technical University of Munich, 81675 Munich, Germany; klaus-peter.janssen@tum.de; 4 II. Medizinische Klinik, Klinikum Rechts der Isar, Technische Universität München, 81675 Munich, Germany; 5 Innere Medizin II, Universitätsklinik Freiburg, Universität Freiburg, 79106 Freiburg, Germany

**Keywords:** esophageal diseases, esophageal carcinogenesis, gut bacteriophages, bacterial exotoxin, LPS biosynthesis proteins

## Abstract

The relationship between viruses (dominated by bacteriophages or phages) and lower gastrointestinal (GI) tract diseases has been investigated, whereas the relationship between gut bacteriophages and upper GI tract diseases, such as esophageal diseases, which mainly include Barrett’s esophagus (BE) and esophageal adenocarcinoma (EAC), remains poorly described. This study aimed to reveal the gut bacteriophage community and their behavior in the progression of esophageal diseases. In total, we analyzed the gut phage community of sixteen samples from patients with esophageal diseases (six BE patients and four EAC patients) as well as six healthy controls. Differences were found in the community composition of abundant and rare bacteriophages among three groups. In addition, the auxiliary metabolic genes (AMGs) related to bacterial exotoxin and virulence factors such as lipopolysaccharides (LPS) biosynthesis proteins were found to be more abundant in the genome of rare phages from BE and EAC samples compared to the controls. These results suggest that the community composition of gut phages and functional traits encoded by them were different in two stages of esophageal diseases. However, the findings from this study need to be validated with larger sample sizes in the future.

## 1. Introduction

Barrett’s esophagus (BE) is the only known precursor for the development of esophageal adenocarcinoma (EAC) with a five-year survival rate of less than 20%. The incidence of these diseases is on the rise globally [1,2]. Early diagnosis of patients at risk could prevent the progression of BE to EAC, and effectively reduce the development of EAC. However, as only 0.3–0.5% of BE patients develop EAC, endoscopic biopsy surveillance, while linked to higher survival rates, is only recommended for at-risk patients [3]. In addition, endoscopies are often discomforting, and sometimes lead to inconclusive results [4]. Thus, noninvasive diagnostics with higher accuracy are sought after. The human gut is home to trillions of microorganisms, including bacteria, viruses, fungi, and protozoa. These microorganisms and their human host maintain a symbiotic relationship, in which the host provides a nutrient-rich habitat, and the microbiota supplies key metabolic capabilities, protects against pathogen invasion, and trains the immune system [5]. In addition, an imbalance in gut microbiota, termed dysbiosis, is associated with several human diseases or conditions, including inflammatory bowel disease (IBD), and colorectal cancer (CRC). These microbial communities have shown disease-specific community structure, suggesting that they can be used as signatures for diagnosing some dysbiosis-associated diseases [6,7,8,9].

Both BE and EAC biopsy samples have been found to harbor a unique bacterial community. Compared to the normal esophagus, Gram-positive bacteria (Firmicutes) were gradually replaced by Gram-negative bacteria (Bacteroidetes, Proteobacteria, Fusobacteria, and Spirochaetes) in BE [10]. As the disease progressed from BE to EAC, the Gram-negative bacteria *Escherichia coli* (*E.coli*) and *Fusobacterium nucleatum* became more dominant [11]. These changes are important as LPS, the outer membrane component of Gram-negative bacteria, could promote the secretion of pro-inflammatory cytokines through activating the Toll-like receptor (TLR) and the downstream NF- κB pathway in different cell types, contributing to the severity of esophageal diseases [11]. In human and mice models with BE, elevated levels of pro-inflammatory cytokines and activated TLR were observed in the gastroesophageal junction [12]. The resulting chronic inflammation could induce systemic immune responses, which further promote the development of GI tract diseases [13]. In the BE mouse model, the chemokines IL-1b and IL-8, secreted by epithelial cells in the esophagus and forestomach squamous epithelium, facilitated the progression of BE to EAC [6]. Moreover, the gut microbiome was associated with this process, as germ-free L2-1L1B mice did not develop dysplasia while the shift of the gut microbiome resulted in different speeds of developing esophageal dysplasia and tumor [6]. The above evidence further shows that these alterations of the bacterial community associated with inflammation can accelerate the development of esophageal diseases.

However, this is not limited to the gut bacteria as viruses, which outnumber bacterial cells by about tenfold in the gut, also contribute to human health and diseases [14,15,16,17,18,19]. In addition to the widely reported eukaryotic viruses [20,21,22,23], mounting data suggests that phages play a critical role in human health by affecting the bacterial community and function [19,24,25]. For example, bacterial-cell lysis caused by phage infection can lead to the release of nucleic acids, proteins, and lipids, which may trigger an inflammation response [26,27]. In addition, prophages that are integrated in bacterial genomes could supply them with virulence-associated genes that can increase their fitness under specific conditions [28]. Under stimulus (such as, DNA damage [29]), the prophages may switch to the lytic cycle [30], which can lead to gene exchange between bacteria, increasing their pathogenicity [31]. For example, the virulence gene that encodes the enterotoxin A was transferred to *Staphylococcus aureus* by phage-mediated horizontal gene transfer (HGT) [32,33]. Furthermore, phages can also obtain AMGs from bacteria to modulate bacterial metabolism [34]. These phage behaviors that regulate bacterial physiology could further indirectly influence human health, such as the occurrence of GI tract and non-GI tract diseases including IBD, CRC, Parkinson’s disease, and Type I diabetes [27,35,36,37].

Former studies that investigated the role of phages in GI tract diseases have mainly focused on the phage community related to lower GI tract diseases, several studies have already described the disease-specific phage community that has been revealed in inflammation-induced diseases such as Crohn’s disease and ulcerative colitis [27]. In a mouse model of intestinal colitis, it was reported that the bacteriophage community structure correlated with the disease status, and the presence of some phages during colitis was associated with an increase in pathobiontic host bacteria (*Escherichia-Shigella*, *Salmonella*, *Mycobacterium*) that was linked to the intestinal inflammation response [38]. However, the role of phages in the upper GI tract remains poorly described and limited to a few studies that have explored the viral community of the oral cavity [39,40]. The research related to the role of the phage community in esophageal diseases is also limited to one study that has used metagenomic data from the whole microbial community without isolating the viral like particles (VLPs) before sequencing [41]. Profiling the community composition of gut phages in esophageal diseases such as BE and EAC can provide some further insight into the role of phages in upper GI tract diseases.

This study aimed to investigate the alteration of gut phages in different stages of esophageal diseases. For this purpose, we (1) determined the composition of the isolated bacteriophage community in BE patients, EAC patients, and healthy controls (CT); (2) predicted the bacterial host ranges of the gut phages in all three groups; (3) identified the metabolic pathways encoded by these phages.

## 2. Materials and Methods

### 2.1. Sample Collection

Sixteen samples were selected from the German BarrettNET registry including six BE patients, four EAC patients, and six CT for virome analysis. The clinical data are shown in Table S1, and additional information can be found in a previous study [42]. Stool samples were collected using Stool Collection Tubes with Stool DNA Stabilizer (STRATEC Molecular GmbH, Berlin, Germany). The sampling procedure was conducted mostly at home or in the clinic if the patients were on outpatient visits. Samples were shipped to the clinic human sample biobank and stored at −80 °C until further virome DNA extraction.

### 2.2. Virome DNA Extraction

The stool samples were vortexed vigorously for 4 h at 4 °C, then centrifuged at 4000 g for 30 min to collect supernatant. The supernatant was passed through 0.22 µm filters (PES Membrane, Lot No. ROCB29300, Merck Millipore, Co., Cork, Ireland) to remove bacterial-associated particles, and the volume was subsequently concentrated to less than 50 µL by Amicon^®^ Ultra Centrifugal Filters (10 kDA, Lot No. R9EA18187, Merck Millipore, Co., Cork, Ireland). Then 1/5 volume of chloroform was mixed with the samples and centrifuged at 14,000 g for 3 min, retaining the upper phase followed by a DNAse I (1 U/µL, Lot No. 1158858, Invitrogen, Carlsbad, CA, USA) treatment for 1 h at 37 °C to remove non-phage DNA. DNase I was inactivated by adding EDTA (0.1M). Subsequently, lysis buffer (700 µL KOH stock (0.43 g/10 mL), 430 µL DDT stock (0.8 g/10 mL), and 370 µL H_2_O, pH = 12) was added to the reaction and incubated at room temperature for 10 min followed by 2 h incubation at −80 °C, and 5 min at 55 °C. Lysed VLPs were then treated for 30 min at 55 °C with Proteinase K (20 mg/mL, Lot No. 1112907, Invitrogen, Carlsbad, CA, USA) to digest remaining viral capsid and extract the virome DNA. AMPure beads (Agencourt, Beckman Coulter, Brea, CA, USA) were added to the extracted DNA and incubated for 15 min at room temperatureF. DNA was eluted from beads by 35 µL Tris buffer (10 mM, pH = 9.8) and stored at −80 °C until it was sent for sequencing. Sequencing was performed on an Illumina HiSeq-PE150 platform.

### 2.3. Bioinformatic Analysis

On average, 9,358,935 ± 169,389 reads per samples were generated. Raw reads were processed with fastp (v0.20.1) [43] to remove adaptors and low-quality bases. Remaining reads were deduplicated using dedupe.sh from bbmap suite (v38.76) (https://sourceforge.net/projects/bbmap/; accessed on 29 January 2020). Then the obtained reads were assembled into contigs using metaSPAdes (v3.14.0) [44] with default parameters retaining only contigs longer than 1 kb. Redundant contigs were removed by dedupe.sh. Remaining contigs were used to predict viral sequences by the combination of VirSorter (v1.0.6) [45], CAT (v5.0.4) [46] and DeepVirFinder (v1.0) [47]. Contigs predicted as category 1 and 2 by Virsorter, or predicted as viruses by CAT, were classified as viruses. Contigs also were classified as viruses if they were predicted as category 3 by VirSorter or could not be classified to taxonomy by CAT but were predicted as a virus by DeepVirFinder with q value < 0.01. Predicted viral contigs were clustered using CD-HIT [48] if they shared >95% identity over 80% of the contig length, the longest contigs in each cluster were retained as a representative for downstream analysis.

For each representative viral contig, ORFs were predicted using Prodigal (v2.6.3) [49] and provided to vConTACT (v2.0) [50] for taxonomy annotation. For contigs that could not be assigned a taxonomy by vConTACT, CAT annotations were used. Otherwise, Order and Family level taxonomic annotations were predicted using Demovir script (https://github.com/feargalr/Demovir; accessed on 27 July 2019) with default parameters and database. To calculate the relative abundances of viruses in each sample, clean reads from each sample were mapped to viral contigs using bbmap.sh from bbmap suite (v38.76). CoverM (v0.4.0) (https://github.com/wwood/CoverM; accessed on 20 February 2020) was used to estimate contig coverage. Feature Counts (v2.0.0) [51] was then used to estimate the number of reads that mapped to each gene. Viral proteins predicted in the previous step were fed into VIBRANT (v1.2.1) [52] to identify lytic and lysogenic phages and the function was annotated using protein mode with default parameters. VIBRANT annotates viral proteins by searching viral proteins against KEGG [53], VOGDB and PFAM databases, which include function annotation of protein sequences and AMGs. The virus (phage)-bacteria (host) interactions were predicted by VirHostMatcher-Net, which is a method based on the combination of features: virus-virus similarity, virus-host alignment-free similarity, virus-host shared CRISPR spacers and virus-host alignment-based matches [54]. Bacterial hosts were predicted for contigs with a length greater than 10 kb and score higher than 95% according to VirHostMatcher-Net.

### 2.4. Statistics Analysis

Alpha diversity of phage community was measured using qiime2 (https://qiime2.org; accessed on 29 January 2020). Principal Coordinates Analysis (PCoA) based on “Bray-Curtis” similarities was performed using R (v3.2, package vegan, The R Foundation, Vienna, Austria, 2016). Permutational Multivariate Analysis of Variance (PERMANOVA) was used to test the significant difference. All data performed statistical analyses, which were conducted in Prism 9- GraphPad (v9.0.0, GraphPad Software, San Diego, CA, USA, 2020) for the two-way analysis of variance [ANOVA], Tukey’s post hoc test, and R (v4.0.2, stats package, The R Foundation, Vienna, Austria, 2020) for the Kruskal–Wallis and Dunn’s post hoc test. The Jonckheere trend test was conducted in IBM SPSS Statistics (v27.0, IBM Corporation, Armonk, NY, USA, 2020). Meanwhile, multiple testing correction were performed to adjust the *p* value based on the “Bonferroni Holm” method. Only significant differences were shown in figures. Graphs were generated using Prism 9- GraphPad (v9.0.0, GraphPad Software, San Diego, CA, USA, 2020), Origin (v2020b, OriginLab Corporation, Northampton, MA, USA, 2020), Microsoft Excel (v365, Microsoft Corporation, Redmond, WA, USA), and R (v3.3.3, ggplot2 package, The R Foundation, Vienna, Austria, 2017). The data in results are provided as average ± SE.

## 3. Results

### 3.1. Gut Bacteriophage Community Structure Differed for BE and EAC Compared to Their Healthy Counterparts

**Figure 1:**
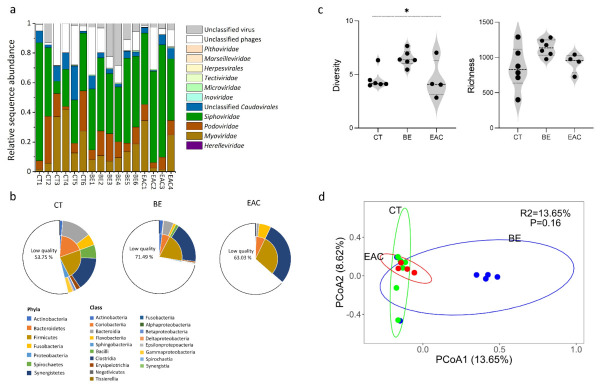
Composition of CT, BE, and EAC VLPs. (**a**) Relative abundance of viral families in CT, BE, and EAC; (**b**) The percentage of predicted bacterial hosts in CT, BE, and EAC. The inner cycle represents bacterial hosts at the phylum level, the outer cycle represents bacterial hosts at the class level. The low quality represents bacterial hosts predicted by contigs with a length lower than 10 kb and the score was lower than 95%; (**c**) Viral alpha diversity including richness (Ace) and diversity (Shannon) in samples from CT, BE, and EAC; (**d**) PCoA plot of the viral community composition based on the Bray–Curtis distances in CT, BE, and EAC samples. CT represents stool samples from healthy controls; BE represents stool samples from Barrett Esophagus patients; EAC represents stool samples from Esophageal Adenocarcinoma patients. Error bars indicate the average ± SE. Statistical significance was determined by Kruskal–Wallis, Dunn’s post hoc test, asterisk indicates *p* < 0.05.

On average, 43 ± 2% of all reads generated through sequencing were from viruses. In total, 854 ± 50, 1136 ± 19, 920 ± 33 viral contigs were obtained from sequences identified as viruses for CT, BE, and EAC, respectively. On average, from these contigs, over 95% of sequences were assigned to phages. The order of *Caudovirales*, which included *Herelleviridae*, *Myoviridae*, *Podoviridae*, *Siphoviridae*, and *Unclassified Caudovirales*, were the most abundant phages, accounting for more than 50% of total sequences in all three groups (Figure 1a, Figure S1). Among those phage families, the relative abundance of *Herelleviridae* was lower than 1% in three groups, the relative abundance of *Myoviridae* (1.12–41.97% in CT, 7.19–18.61% in BE, 1.37–34.36% in EAC), *Podoviridae* (2.03–31.68% in CT, 5.72–18.44% in BE, 3.72–11.01% in EAC) and *Siphoviridae* (8.28–79.60% in CT, 36.89–57.19% in BE, 41.48–75.69% in EAC) showed great variation within each group (*p* > 0.05). Some viral contigs were assigned to other phage or viral families including *Inoviridae*, *Microviridae*, *Tectiviridae*, *Herpesvirales*, *Marseilleviridae*, and *Pithoviridae* with a relative abundance of less than 1%. Meanwhile, the large difference in specific viral taxa between individuals was observed in the same group, which may be attributable to multiple factors such as age, gender, diet, or drug usage (Table S1). We next determined the dominant phage replication cycle (lytic versus lysogenic cycle). On average, EAC samples had more temperate phages (lysogenic cycle) than BE and CT (*p* > 0.05), 11.97% ± 2.43% in CT, 13.47% ± 1.15% in BE, 19.13% ± 4.90% in EAC (Figure S2).

We next predicted the bacterial host range of the viral contigs from different groups in the study (Figure 1b). We observed that the bacterial hosts mainly spanned the phyla *Actinobacteria*, *Bacteroidetes*, *Firmicutes*, and *Proteobacteria*, which were common across all three groups. In addition, we found that less than 0.1% of the phages were predicted to infect *Fusobacteria*, *Spirochaetes*, and *Synergistetes*. When the predicted bacterial host in class level was further compared, their relative abundance showed more obvious variation among the different groups, but these results were not statistically significant. For *Actinobacteria*, the relative abundance in CT (1.33% ± 0.28%) and BE (1.77% ± 0.21%) was higher than EAC (0.37% ± 0.11%) (*p* > 0.05). For *Flavobacteriia*, the relative abundance in CT (5.02% ± 1.45%) and EAC (5.38% ± 1.45%) was higher than BE (1.14% ± 0.16%) (*p* > 0.05). Notably, the classes *Betaproteobacteria*, *Deltaproteobacteria*, and *Gammaproteobacteria* were more abundant in CT compared with BE and EAC. Moreover, the relative abundance of *Bacteroidia* (13.08% ± 2.34% in CT, 4.38% ± 0.45% in BE, 1.25% ± 0.22% in EAC), *Bacilli* (5.97% ± 1.51% in CT,1.33% ± 0.14% in BE, <0.1% in EAC), and *Erysipelotrichia* (1.86% ± 0.54% in CT, 0.65% ± 0.093% in BE, <0.1% in EAC) were lower in BE and EAC compared to CT, while the relative abundance of *Clostridia* (15.06% ± 0.52% in CT, 18.04% ± 0.90% in BE, 29.20% ± 5.60% in EAC) was higher in BE and EAC compared to CT. However, there was no significant difference (Jonckheere trend test, *p* > 0.05). Furthermore, the remaining classes had a lower relative abundance (0.0001%–0.31%) across the three groups.

We further examined how the changes in phages community composition affected the overall diversity. For the alpha diversity, a significant difference in phage diversity (Shannon) was found among the three groups (*p* = 0.036), while no significant difference was observed in phage richness (Ace) (*p* > 0.05) (Figure 1c). Furthermore, the alpha diversity showed differences among BE and EAC compared to CT samples (*p* > 0.05). Specifically, in both BE (1136.17 ± 19.48) and EAC (920.50 ± 33.87), the richness (Ace) was higher compared with that in CT (854.00 ± 50.73). However, only in BE (6.50 ± 0.11), the diversity (Shannon) was higher compared with that in CT (4.53 ± 0.15). Furthermore, BE had a higher level of richness (Ace) and diversity (Shannon) than EAC. In addition, no significant difference was detected (*p* > 0.05) in beta diversity (PCoA) among the three groups (Figure 1d).

### 3.2. Abundant and Rare Phage Communities in the Gut May Contribute to the Progress of Esophageal Carcinogesis

**Figure 2:**
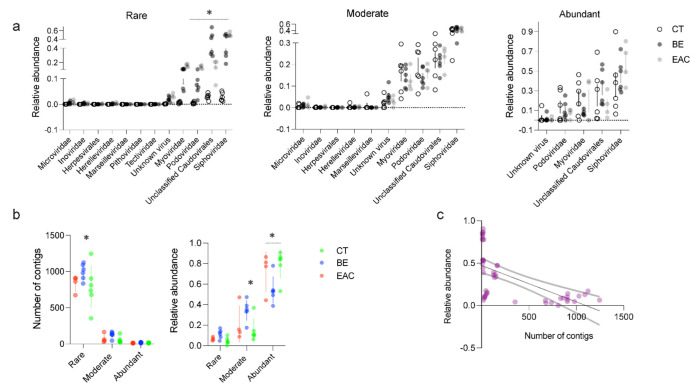
Composition of the rare, moderate, and abundant gut viruses in CT, BE and EAC samples. Rare, moderate, and abundant viruses were categorized based on the viral contig level. Abundant viruses represent viral contigs whose relative abundance was more than 1% in total contigs, moderate viruses represent viral contigs whose relative abundance was more than 0.1% and less than 1% in total contigs, and rare viruses represent viral contigs whose relative abundance was less than 0.1% in total contigs. (**a**) The relative abundance of viral families; (**b**) Number of contigs generated each viral contig category, rare, moderate, and abundant, on left and relative abundance of them on right. (**c**) Negative correlation between number of contigs, from rare, moderate, and abundant phages, and their relative abundance. CT represents stool samples from healthy controls; BE represents stool samples from Barrett Esophagus patients; EAC represents stool samples from Esophageal Adenocarcinoma patients. Statistical significance was determined by two–way analysis of variance [ANOVA], Tukey’s post hoc test, asterisk indicates *p* < 0.05.

We used a sorting approach commonly applied in ecological study that classifies microbes into three groups based on their abundance [55,56], aiming to explore the role of less abundant microbes in different ecosystems. Using this approach, the contribution of rare, less abundant, bacterial Operational Taxonomic Units (OTUs) to some of the key ecological functions was revealed in the environment [57], which was previously overlooked. We believe this approach can be beneficial for studying phages in the gut. To this end, we divided phage contigs into abundant phages (relative abundance was more than 1% in total viral contigs), moderate phages (relative abundance was more than 0.1% and less than 1% in total viral contigs), and rare phages (relative abundance was less than 0.1% in total viral contigs). At these three relative abundance levels, members of the order *Caudovirales* (*Myoviridae, Siphoviridae*, and *Podoviridae*) showed the highest relative abundance in all three groups (Figure 2a). Subsequently, we observed that abundant phages presented significantly higher relative abundance (79.54% ± 2.28% in CT, 54.28% ± 2.19% in BE, and 72.25% ± 4.06% in EAC) when compared with moderate (14.79% ± 1.83% in CT, 34.38% ± 1.68% in BE, and 21.19% ± 3.57% in EAC) and rare phages (4.51% ± 0.52% in CT, 11.34% ± 0.85% in BE, and 6.56% ± 0.52% in EAC) in all three groups (abundant vs. moderate *p* < 0.001, abundant vs rare *p* < 0.001) (Figure 2b,c), while the highest number of contigs was from rare phages (788 ± 48 in CT, 994 ± 18 in BE, and 836 ± 28 in EAC), exceeding abundant (13 ± 1 in CT, 17 ± 1 in BE, and 11 ± 1 in EAC) and moderate (54 ± 8 in CT, 126 ± 7 in BE, and 74 ± 15 in EAC) phages in all three groups (Figure 2b,c). The highest relative abundance of abundant phages and the highest number of contigs of rare phages may suggest their different behaviors in relation to the gut bacterial community and esophageal diseases. Moreover, a significant difference was observed in beta-diversity on abundant (*p* = 0.004) and rare phages (*p* = 0.003) (Figure S3), which may imply that these two groups of phages showed higher sensitivity to the changes in the upper GI tract through esophageal disease progression. In addition, we found that the abundance of temperate phages that displayed a lysogenic replication cycle increased with the development of esophageal diseases. This may suggest a higher occurrence of HGT in these samples.

To further evaluate the importance of rare phages in HGT, we compared these three groups of phages to the number of bacterial hosts they infect. On the class level, we observed small differences between phage groups from different health conditions, rare phages infected 18 different bacterial classes whereas abundant phages infected 14 (Figure 1c). However, when bacterial hosts were compared on the genus level, both diversity and abundance showed large differences, 84 for rare versus 46 for abundant phages (Table S2). In particular, contigs belonging to rare phages showed similar characteristics regarding the number of hosts they infect over three groups, showing a broader bacterial host range compared to moderate and abundant phages. For example, the contigs from rare phages were able to infect 6 or 7 different bacterial hosts at the genus level (Table S3), which was relatively higher than the bacterial hosts predicted for the contigs from abundant and moderate phages. The broader bacterial host range and higher number of contigs (Figure 2b, Tables S2 and S3) of rare phages could potentially lead to storing more AMGs in their genomes and, in turn, expand the frequency of HGT between gut bacteria.

### 3.3. AMGs Found in Rare Bacteriophages Showed Increment in Esophageal Diseases

**Figure 3:**
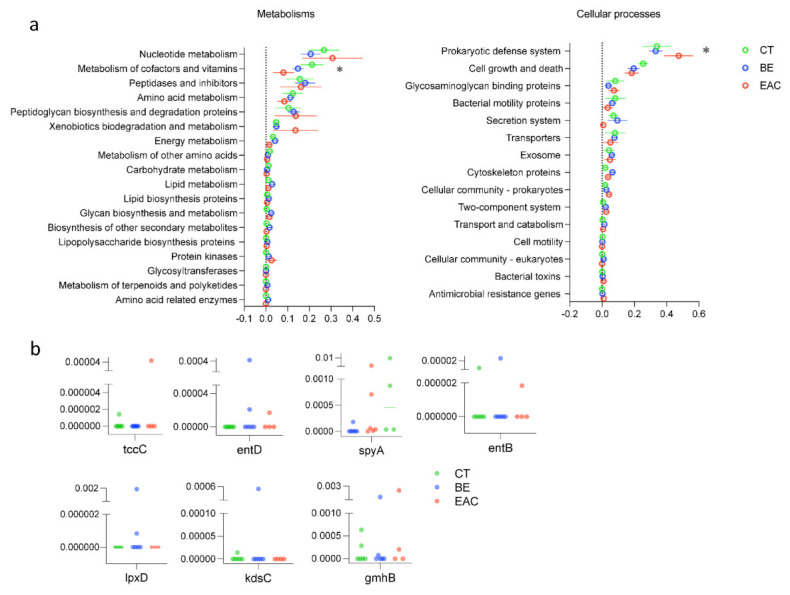
Viral functional traits. (**a**) The relative abundance of different functional traits in viral sequences; (**b**) The relative abundance of genes encoding four different bacterial toxins with higher abundance in BE and EAC samples compared with CT on the top, and genes encoding the LPS biosynthesis proteins on the bottom. Error bars indicate the average ± SE. Statistical significance was determined by two–way analysis of variance [ANOVA], Tukey’s post hoc test, asterisk indicates *p* < 0.05.

After annotation of the viral contigs, viruses were found to be involved in most of the microbial functions related to metabolism, cellular processes, genetic information processing, environment information processing, organismal system, and human disease (Figure 3a and Figure S4). Significant differences were found for genes related to metabolism of cofactors and vitamins (*p* = 0.0083) and genes related to the prokaryotic defense system among the three groups (*p* = 0.0202) (Figure 3a). Genes involved in metabolism of cofactors and vitamins were found to be most abundant in CT phages, whereas genes related to the prokaryotic defense system were more abundant in EAC phages, suggesting a stronger arms race between phages and bacteria in this disease (Figure 3a). Notably, AMGs encoding bacterial toxins were found to be more abundant in the genome of rare bacteriophages including the *spyA* gene, *tccC* gene, *entB* gene and *entD* gene, which are involved in microbial cellular processes. The *spyA* gene, which encodes a C3 family ADP-ribosyltransferase (bacterial exotoxin) [58], showed a slightly higher level of relative abundance in BE and EAC (*p* > 0.05) compared with the other three AMGs (Figure 3b). Moreover, the *spyA* gene level was relatively higher in BE (0.00040 ± 0.00011) and EAC (0.0027 ± 0.0012) compared with CT (0.00031 ± 0.000012) (*p* > 0.05). Other AMGs that relate to LPS biosynthesis proteins were also found in the genome of rare phages including the *lpxD* gene, *kdsC* gene and *gmnB* gene, which are involved in microbial metabolism (Figure 3b). The *lpxD* gene only presented in BE with a relative abundance of 0.00031 ± 0.000113. The *kdsC* gene presented in BE (0.000089 ± 0.000036) and CT (0.0000024 ± 0.00000097). For the *gmnB* gene, it was relatively higher in EAC (0.00064 ± 0.00029) and BE (0.00024 ± 0.000094) compared with CT (0.00015 ± 0.000044) (*p* > 0.05). The higher abundance of these genes in phages from BE and EAC compared to CT may have resulted from the increase of pathogenic bacteria, mainly Gram-negatives, in the esophageal diseases, leading to a higher chance of obtaining AMGs, which are related to LPS biosynthesis proteins encoded by phages. We next explored the appearance of these genes in the Gut Phages Database (GPD) containing 142,809 non-redundant globally distributed phage genomes. We found many phages encoding these genes in GPD with one exception, *tccC*, showing these AMGs are ubiquitous in the human gut (Figure S5). Toxin complex (Tc) is a multisubunit toxin consisting of three components (TcA, B, and C) encoded by pathogenic bacteria infecting both insects and humans. TcAs that make functional pores combine with TcB-TcC subunits to create active chimeric holotoxins. Tc toxins are encoded by human pathogens like *Yersinia pestis*, *Y. pseudotuberculosis*, and *Morganella morganii* and are believed to significantly contribute to these bacteria’s pathogenicity. Yet, their role in EAC remains to be revealed [59]. The increase of these genes in phages from BE and EAC may contribute to the severity of these diseases through exchanging genes that are involved in bacterial exotoxin production and LPS biosynthesis in esophageal carcinogenesis. This warrants further investigation.

## 4. Discussion

Barrett’s esophagus (BE) is a condition caused by the metaplastic replacement of the normal squamous epithelium by columnar epithelium. BE is closely associated with the development of esophageal adenocarcinoma (EAC), a disease in which cancerous cells develop in the tissues of the esophagus with a high mortality rate [42]. It has been recently shown that gut dysbiosis can activate oncogenic signaling pathways, leading to the production of tumor-promoting metabolites, and further influence the esophageal mucosal inflammation and tumorigenesis [60]. For example, gut bacteria regulate bile acid (BA) metabolism. Under stimulation such as a high-fat diet, the gut bacteria changed, and the level of BA increased accordingly [61]. The reflux of BA to the esophagus caused esophageal damage, leading to BE and subsequent EAC. In an animal experiment simulating BA reflux, overexpression of the inflammatory cells, IL-6 and TNF- α, was found [62]. This indicated that gut bacterial alterations could indirectly induce the esophageal mucosal inflammation and carcinogenesis [62,63,64]. Despite a wealth of data on the role of gut bacteria in GI tract disease, we have only recently recognized the association of gut viruses with some GI tract diseases, including CRC in which the diversity of the gut viruses is significantly increased in stool samples from CRC patients, suggesting a disease-specific signature that can be used to differentiate CRC samples from controls [37]. The CRC-associated virome includes primarily temperate bacteriophages belonging to *Siphoviridae* and *Myoviridae* families [65]. The impact of phages on gut homeostasis is not restricted to their interactions with gut bacteria as phages can directly interact with the human host. In vitro studies have demonstrated that phages can cross the epithelial cell layer through transcytosis, thereby stimulating the underlying immune cells [22,66,67,68,69]. For example, the interaction between *E.coli* phages and the immune system has been associated with Type I Diabetes autoimmunity [36]. It has been reported that phages can activate IFN-γ produced by CD4+ T cells via the nucleotide-sensing receptor TLR9, which accelerates intestinal inflammation and colitis, leading to a systemic inflammation response [70]. The consistent disease-specific signature of gut viruses [27,37], suggests a potential association between gut viruses and human disease.

Studies that investigated the esophageal virome, using metagenomic data of whole microbial communities rather than profiling the isolated viral communities, have identified a range of phages, including *Streptococcus*, *Campylobacter*, *Lactococcus*, and *γ*-Proteobacteria phages [71]. The aforementioned and those that only explored the bacterial community of the esophagus have mainly used biopsy samples for virome and bacterium analysis [10,72,73]. Although, biopsies could directly reflect the disease-associated microbial signature at the lesion, the sampling procedure is invasive, time-consuming, costly, and may induce potential complications [74]. Moreover, biopsy samples often have limited microbial materials, with a lower probability of successful sequencing and downstream analysis [75]. Thus, an amplification step (e.g., whole genome amplification) is necessary, which might introduce biases to study results. On the contrary, stool samples collected by non-invasive methods often supply sufficient materials for research purposes [76].

Here we explored stool samples from BE, EAC, and CT phages community composition in esophageal diseases. Our in-depth gut virome analysis during esophageal carcinogenesis provided some evidence of gut phage community changes between different stages of esophageal diseases. Consistent with previous studies that have explored the gut viruses, mainly in the lower GI tract diseases such as IBD and CRC [27,65], phages from the order *Caudovirales* were the most dominant phages in the samples from esophageal diseases. Compared with CT, the alpha diversity has changed with the esophageal diseases progress, and a relatively higher alpha diversity was observed in BE samples compared to CT and EAC. This was not reflected in the beta diversity as no significant differences were observed among three groups. Using a common sorting approach in microbial ecology, we identified disease-associated differences in diversity and abundance of rare phages, suggesting a potential link between these phages and esophageal diseases. In addition, consistent with previous studies on diseases like IBD [77] and CRC [65], we observed changes in the proportion of lytic/lysogenic replication cycles of phages, and more temperate phages were observed in esophageal carcinogenesis. These results further support earlier studies that reported the dominance of virulent phages (lytic cycle) in the healthy human gut replaced by temperate phages in Crohn’s disease and ulcerative colitis [23,24]. Furthermore, the relatively higher percentage of temperate phages in samples from esophageal diseases may imply more influence on the bacterial physiology through phage mediated HGT in those groups. However, we did not study the bacterial community of these samples, the community structure of the predicted bacterial hosts for the phages identified in the study may suggest a complex relationship between bacteria and bacteriophage community in esophageal diseases. Earlier studies on lower GI tract diseases such as CRC have observed that the effect of phages resulted from their interactions with the whole bacterial community, rather than the bacterial taxa directly contributing to the disease severity [65]. However, there was no direct correlation between bacterial diversity and phage diversity [27,37].

In addition, we found several AMGs in the genome of the rare phages, further emphasizing the potential role of phages in regulating bacterial physiology by supplying their host with beneficial genes. Specifically, a slightly higher abundance of *spyA* (*p* > 0.05) was observed in BE and EAC, potentially contributing to the production of bacterial exotoxins, which disrupt cytoskeletal structures and promote colonization of pathogenic bacteria [58]. The relatively higher abundance of AMGs related to LPS biosynthesis proteins were also found in BE and EAC, which may indicate the dominance of Gram-negative bacteria and the potential inflammatory effects of phage–bacteria interactions. Phages that carry these AMGs can introduce these genes to the genome of gut bacteria via integration, which may contribute to the severity of the esophageal diseases through lysogenic conversion. This could further induce gut inflammation through expression of the phage-derived virulence genes and deteriorate esophageal disease. Intestinal permeability caused by phage-mediated changes of gut microbiota could also lead to systemic inflammatory responses [78]. Given the high variability of the microbiome between individuals and the limited number of samples analyzed, it is difficult to identify significant differences in viral community structure between different groups in the current study. Thus, our findings should be further pursued with a larger sample size.

## 5. Conclusions

In summary, this study provides further evidence of potential relationship between gut phages and esophageal diseases. Interestingly, the distinct gut phage community structure was identified in two different stages of esophageal diseases, and these differences were mainly found in abundant and rare bacteriophages. Notably, rare phages and HGT mediated by them have been found to be more related to esophageal diseases. Specially, the rare phages contributed to enriching AMGs related to bacterial exotoxin and LPS biosynthesis proteins, and the possible upregulated level of these genes. These, in turn, may contribute to changes in the gut bacterial composition and inflammation, which lead to the development of esophageal diseases, as previously suggested [6]. However, given the small sample size in our study, the potential diagnostic importance of AMGs and disease-specific viral signature identified should be experimentally validated in further studies.

## Data Availability

The data presented in this study are openly available in NCBI Sequence Read Archive (SRA) under accession number SUB8621833.

## Supplementary Materials

The following are available online at https://www.mdpi.com/article/10.3390/microorganisms9081701/s1, Figure S1: Gene-sharing taxonomic network of viral sequences in this study, including viral RefSeq viruses v85. Figure S2: The proportion of lytic/lysogenic replication cycles predicted for the viral contigs from three groups. Figure S3: PCoA plot of the viral community composition based on the Bray-Curtis distances in CT, BE, and EAC samples. Figure S4: The relative abundance of different functional traits in viral sequences. Figure S5: The number of phages that contained the identified AMGs of this study in the Gut Phage Database (GPD). Table S1: Clinical information of individuals from three groups. Table S2: The relative abundance of bacterial host at genus level for abundant, moderate, and rare bacteriophages. Table S3: The percentage of contig relative abundance in different number of predicted bacterial genus types for abundant, moderate, and rare bacteriophages. Table S4: The relative abundance of identified AMGs.

## References

[1] He Y., Li D., Shan B., Liang D., Shi J., Chen W., He J. (2019). Incidence and mortality of esophagus cancer in China, 2008–2012. Chin. J. Cancer Res..

[2] Pennathur A., Gibson M.K., Jobe B.A., Luketich J.D. (2013). Oesophageal carcinoma. Lancet.

[3] Martinucci I., De Bortoli N., Russo S., Bertani L., Furnari M., Mokrowiecka A., Malecka-Panas E., Savarino V., Savarino E., Marchi S. (2016). Barrett’s esophagus in 2016: From pathophysiology to treatment. World J. Gastrointest. Pharmacol. Ther..

[4] Pohl H., Koch M., Khalifa A., Papanikolaou I., Scheiner K., Wiedenmann B., Rösch T. (2007). Evaluation of endocytoscopy in the surveillance of patients with Barrett’s esophagus. Endoscopy.

[5] Hooper L.V., Gordon J.I. (2001). Commensal host-bacterial relationships in the gut. Science.

[6] Münch N.S., Fang H.-Y., Ingermann J., Maurer H.C., Anand A., Kellner V., Sahm V., Wiethaler M., Baumeister T., Wein F. (2019). High-fat diet accelerates carcinogenesis in a mouse model of barrett’s esophagus via interleukin 8 and alterations to the gut microbiome. Gastroenterology.

[7] Snider E.J., Compres G., Freedberg D.E., Khiabanian H., Nobel Y.R., Stump S., Uhlemann A.-C., Lightdale C.J., Abrams J.A. (2019). Alterations to the esophageal microbiome associated with progression from Barrett’s esophagus to esophageal adenocarcinoma. Cancer Epidemiol. Prev. Biomark..

[8] Ren Z., Li A., Jiang J., Zhou L., Yu Z., Lu H., Xie H., Chen X., Shao L., Zhang R. (2019). Gut microbiome analysis as a tool towards targeted non-invasive biomarkers for early hepatocellular carcinoma. Gut.

[9] Manor O., Dai C.L., Kornilov S.A., Smith B., Price N.D., Lovejoy J.C., Gibbons S.M., Magis A.T. (2020). Health and disease markers correlate with gut microbiome composition across thousands of people. Nat. Commun..

[10] Yang L., Lu X., Nossa C.W., Francois F., Peek R.M., Pei Z. (2009). Inflammation and intestinal metaplasia of the distal esophagus are associated with alterations in the microbiome. Gastroenterology.

[11] Yang L., Francois F., Pei Z. (2012). Molecular pathways: Pathogenesis and clinical implications of microbiome alteration in esophagitis and Barrett esophagus. Clin. Cancer Res..

[12] Zaidi A.H., Kelly L.A., Kreft R.E., Barlek M., Omstead A.N., Matsui D., Boyd N.H., Gazarik K.E., Heit M.I., Nistico L. (2016). Associations of microbiota and toll-like receptor signaling pathway in esophageal adenocarcinoma. BMC Cancer.

[13] Okereke I., Hamilton C., Wenholz A., Jala V., Giang T., Reynolds S., Miller A., Pyles R. (2019). Associations of the microbiome and esophageal disease. J. Thorac. Dis..

[14] Wylie K.M., Weinstock G.M., Storch G.A. (2012). Emerging view of the human virome. Transl. Res..

[15] Carding S.R., Davis N., Hoyles L. (2017). The human intestinal virome in health and disease. Aliment. Pharmacol. Ther..

[16] Cadwell K. (2015). The virome in host health and disease. Immunity.

[17] Lepage P., Leclerc M.C., Joossens M., Mondot S., Blottière H.M., Raes J., Ehrlich D., Doré J. (2013). A metagenomic insight into our gut’s microbiome. Gut.

[18] Mills S., Shanahan F., Stanton C., Hill C., Coffey A., Ross R.P. (2013). Movers and shakers: Influence of bacteriophages in shaping the mammalian gut microbiota. Gut Microbes.

[19] Dalmasso M., Hill C., Ross R.P. (2014). Exploiting gut bacteriophages for human health. Trends Microbiol..

[20] Ungaro F., Massimino L., Furfaro F., Rimoldi V., Peyrin-Biroulet L., D’alessio S., Danese S. (2019). Metagenomic analysis of intestinal mucosa revealed a specific eukaryotic gut virome signature in early-diagnosed inflammatory bowel disease. Gut Microbes.

[21] Tetz G., Tetz V. (2018). Prion-like domains in eukaryotic viruses. Sci. Rep..

[22] Breitbart M., Hewson I., Felts B., Mahaffy J.M., Nulton J., Salamon P., Rohwer F. (2003). Metagenomic analyses of an uncultured viral community from human feces. J. Bacteriol..

[23] Reyes A., Haynes M., Hanson N., Angly F.E., Heath A.C., Rohwer F., Gordon J.I. (2010). Viruses in the faecal microbiota of monozygotic twins and their mothers. Nature.

[24] Sabino J., Hirten R.P., Colombel J.F. (2020). bacteriophages in gastroenterology—From biology to clinical applications. Aliment. Pharmacol. Ther..

[25] De Sordi L., Lourenço M., Debarbieux L. (2019). The battle within: Interactions of bacteriophages and bacteria in the gastrointestinal tract. Cell Host Microbe.

[26] Łusiak-Szelachowska M., Weber-Dąbrowska B., Jończyk-Matysiak E., Wojciechowska R., Górski A. (2017). Bacteriophages in the gastrointestinal tract and their implications. Gut Pathog..

[27] Norman J.M., Handley S.A., Baldridge M.T., Droit L., Liu C.Y., Keller B.C., Kambal A., Monaco C.L., Zhao G., Fleshner P. (2015). Disease-specific alterations in the enteric virome in inflammatory bowel disease. Cell.

[28] Mirzaei M.K., Xue J., Costa R., Ru J., Schulz S., Taranu Z.E., Deng L. (2021). Challenges of studying the human virome–relevant emerging technologies. Trends Microbiol..

[29] Lin E.C., Lynch A.S. (2012). Regulation of Gene Expression in Escherichia coli.

[30] Feiner R., Argov T., Rabinovich L., Sigal N., Borovok I., Herskovits A.A. (2015). A new perspective on lysogeny: Prophages as active regulatory switches of bacteria. Nat. Rev. Microbiol..

[31] Brüssow H., Canchaya C., Hardt W.-D. (2004). Phages and the evolution of bacterial pathogens: From genomic rearrangements to lysogenic conversion. Microbiol. Mol. Biol. Rev..

[32] Saunders J.R., Allison H., James C.E., McCarthy A.J., Sharp R. (2001). Phage-mediated transfer of virulence genes. J. Chem. Technol. Biotechnol..

[33] Coleman D.C., Sullivan D.J., Russel R.J., Arbuthnott J.P., Carey B.F., Pomeroy H.M. (1989). Staphylococcus aureus bacteriophages mediating the simultaneous lysogenic conversion of β-lysin, staphylokinase and enterotoxin A: Molecular mechanism of triple conversion. Microbiology.

[34] Crummett L.T., Puxty R.J., Weihe C., Marston M.F., Martiny J.B. (2016). The genomic content and context of auxiliary metabolic genes in marine cyanomyoviruses. Virology.

[35] Tetz G., Brown S.M., Hao Y., Tetz V. (2018). Parkinson’s disease and bacteriophages as its overlooked contributors. Sci. Rep..

[36] Tetz G., Brown S.M., Hao Y., Tetz V. (2019). Type 1 diabetes: An association between autoimmunity, the dynamics of gut amyloid-producing E. coli and their phages. Sci. Rep..

[37] Nakatsu G., Zhou H., Wu W.K.K., Wong S.H., Coker O.O., Dai Z., Li X., Szeto C.-H., Sugimura N., Lam T.Y.-T. (2018). Alterations in enteric virome are associated with colorectal cancer and survival outcomes. Gastroenterology.

[38] Duerkop B.A., Kleiner M., Paez-Espino D., Zhu W., Bushnell B., Hassell B., Winter S.E., Kyrpides N.C., Hooper L.V. (2018). Murine colitis reveals a disease-associated bacteriophage community. Nat. Microbiol..

[39] Ly M., Abeles S.R., Boehm T.K., Robles-Sikisaka R., Naidu M., Santiago-Rodriguez T., Pride D.T. (2014). Altered oral viral ecology in association with periodontal disease. MBio.

[40] Abeles S.R., Robles-Sikisaka R., Ly M., Lum A.G., Salzman J., Boehm T.K., Pride D.T. (2014). Human oral viruses are personal, persistent and gender-consistent. ISME J..

[41] Deshpande N.P., Riordan S.M., Castaño-Rodríguez N., Wilkins M.R., Kaakoush N.O. (2018). Signatures within the esophageal microbiome are associated with host genetics, age, and disease. Microbiome.

[42] Wiethaler M., Slotta-Huspenina J., Brandtner A., Horstmann J., Wein F., Baumeister T., Radani N., Gerland S., Anand A., Lange S. (2019). BarrettNET—A prospective registry for risk estimation of patients with Barrett’s esophagus to progress to adenocarcinoma. Dis. Esophagus.

[43] Chen S., Zhou Y., Chen Y., Gu J. (2018). fastp: An ultra-fast all-in-one FASTQ preprocessor. Bioinformatics.

[44] Nurk S., Meleshko D., Korobeynikov A., Pevzner P.A. (2017). metaSPAdes: A new versatile metagenomic assembler. Genome Res..

[45] Roux S., Enault F., Hurwitz B.L., Sullivan M.B. (2015). VirSorter: Mining viral signal from microbial genomic data. PeerJ.

[46] von Meijenfeldt F.B., Arkhipova K., Cambuy D.D., Coutinho F.H., Dutilh B.E. (2019). Robust taxonomic classification of uncharted microbial sequences and bins with CAT and BAT. Genome Biol..

[47] Ren J., Song K., Deng C., Ahlgren N.A., Fuhrman J.A., Li Y., Xie X., Poplin R., Sun F. (2020). Identifying viruses from metagenomic data using deep learning. Quant. Biol..

[48] Fu L., Niu B., Zhu Z., Wu S., Li W. (2012). CD-HIT: Accelerated for clustering the next-generation sequencing data. Bioinformatics.

[49] Hyatt D., Chen G.-L., LoCascio P.F., Land M.L., Larimer F.W., Hauser L.J. (2010). Prodigal: Prokaryotic gene recognition and translation initiation site identification. BMC Bioinform..

[50] Jang H.B., Bolduc B., Zablocki O., Kuhn J.H., Roux S., Adriaenssens E.M., Brister J.R., Kropinski A.M., Krupovic M., Lavigne R. (2019). Taxonomic assignment of uncultivated prokaryotic virus genomes is enabled by gene-sharing networks. Nat. Biotechnol..

[51] Liao Y., Smyth G.K., Shi W. (2014). featureCounts: An efficient general purpose program for assigning sequence reads to genomic features. Bioinformatics.

[52] Kieft K., Zhou Z., Anantharaman K. (2020). VIBRANT: Automated recovery, annotation and curation of microbial viruses, and evaluation of viral community function from genomic sequences. Microbiome.

[53] Kanehisa M., Furumichi M., Tanabe M., Sato Y., Morishima K. (2017). KEGG: New perspectives on genomes, pathways, diseases and drugs. Nucleic Acids Res..

[54] Wang W., Ren J., Tang K., Dart E., Ignacio-Espinoza J.C., Fuhrman J.A., Braun J., Sun F., Ahlgren N.A. (2020). A network-based integrated framework for predicting virus–prokaryote interactions. NAR Genom. Bioinform..

[55] Ji M., Kong W., Stegen J., Yue L., Wang F., Dong X., Cowan D.A., Ferrari B.C. (2020). Distinct assembly mechanisms underlie similar biogeographical patterns of rare and abundant bacteria in Tibetan Plateau grassland soils. Environ. Microbiol..

[56] Sjöstedt J., Koch-Schmidt P., Pontarp M., Canbäck B., Tunlid A., Lundberg P., Hagström Å., Riemann L. (2012). Recruitment of members from the rare biosphere of marine bacterioplankton communities after an environmental disturbance. Appl. Environ. Microbiol..

[57] Vigneron A., Cruaud P., Alsop E., de Rezende J.R., Head I.M., Tsesmetzis N. (2018). Beyond the tip of the iceberg; a new view of the diversity of sulfite-and sulfate-reducing microorganisms. ISME J..

[58] Coye L.H., Collins C.M. (2004). Identification of SpyA, a novel ADP-ribosyltransferase of Streptococcus pyogenes. Mol. Microbiol..

[59] Leidreiter F., Roderer D., Meusch D., Gatsogiannis C., Benz R., Raunser S. (2019). Common architecture of Tc toxins from human and insect pathogenic bacteria. Sci. Adv..

[60] Deng Y., Tang D., Hou P., Shen W., Li H., Wang T., Liu R. (2021). Dysbiosis of gut microbiota in patients with esophageal cancer. Microb. Pathog..

[61] Schwabe R.F., Jobin C. (2013). The microbiome and cancer. Nat. Rev. Cancer.

[62] Sun D., Wang X., Gai Z., Song X., Jia X., Tian H. (2015). Bile acids but not acidic acids induce Barrett’s esophagus. Int. J. Clin. Exp. Pathol..

[63] Schmidt M., Ankerst D.P., Chen Y., Wiethaler M., Slotta-Huspenina J., Becker K.-F., Horstmann J., Kohlmayer F., Lehmann A., Linkohr B. (2020). Epidemiologic Risk Factors in a Comparison of a Barrett Esophagus Registry (BarrettNET) and a Case–Control Population in Germany. Cancer Prev. Res..

[64] Elliott D.R.F., Perner J., Li X., Symmons M.F., Verstak B., Eldridge M., Bower L., O’Donovan M., Gay N.J., Fitzgerald R.C. (2017). Impact of mutations in Toll-like receptor pathway genes on esophageal carcinogenesis. PLoS Genet..

[65] Hannigan G.D., Duhaime M.B., Ruffin M.T., Koumpouras C.C., Schloss P.D. (2018). Diagnostic potential and interactive dynamics of the colorectal cancer virome. MBio.

[66] Sinha A., Maurice C.F. (2019). Bacteriophages: Uncharacterized and dynamic regulators of the immune system. Mediat. Inflamm..

[67] Nguyen S., Baker K., Padman B.S., Patwa R., Dunstan R.A., Weston T.A., Schlosser K., Bailey B., Lithgow T., Lazarou M. (2017). Bacteriophage transcytosis provides a mechanism to cross epithelial cell layers. MBio.

[68] Bichet M.C., Chin W.H., Richards W., Lin Y.-W., Avellaneda-Franco L., Hernandez C.A., Oddo A., Chernyavskiy O., Hilsenstein V., Neild A. (2021). Bacteriophage uptake by mammalian cell layers represents a potential sink that may impact phage therapy. Iscience.

[69] Wahida A., Tang F., Barr J.J. (2021). Rethinking phage-bacteria-eukaryotic relationships and their influence on human health. Cell Host Microbe.

[70] Gogokhia L., Buhrke K., Bell R., Hoffman B., Brown D.G., Hanke-Gogokhia C., Ajami N.J., Wong M.C., Ghazaryan A., Valentine J.F. (2019). Expansion of bacteriophages is linked to aggravated intestinal inflammation and colitis. Cell Host Microbe.

[71] Moonen A., Annese V., Belmans A., Bredenoord A.J., Des Varannes S.B., Costantini M., Dousset B., Elizalde J.I., Fumagalli U., Gaudric M. (2016). Long-term results of the European achalasia trial: A multicentre randomised controlled trial comparing pneumatic dilation versus laparoscopic Heller myotomy. Gut.

[72] Pei Z., Bini E.J., Yang L., Zhou M., Francois F., Blaser M.J. (2004). Bacterial biota in the human distal esophagus. Proc. Natl. Acad. Sci. USA.

[73] Pei Z., Yang L. (2005). Bacterial biota in reflux esophagitis and Barrett’s esophagus. World J. Gastroenterol..

[74] Fillon S.A., Harris J.K., Wagner B.D., Kelly C.J., Stevens M.J., Moore W., Fang R., Schroeder S., Masterson J.C., Robertson C.E. (2012). Novel device to sample the esophageal microbiome—The esophageal string test. PLoS ONE.

[75] Lim E.H., Zhang S.-L., Li J.-L., Yap W.-S., Howe T.-C., Tan B.-P., Lee Y.-S., Wong D., Khoo K.-L., Seto K.-Y. (2009). Using whole genome amplification (WGA) of low-volume biopsies to assess the prognostic role of EGFR, KRAS, p53, and CMET mutations in advanced-stage non-small cell lung cancer (NSCLC). J. Thorac. Oncol..

[76] Bull M.J., Plummer N.T. (2014). Part 1: The human gut microbiome in health and disease. Integr. Med. A Clin. J..

[77] Clooney A.G., Sutton T.D., Shkoporov A.N., Holohan R.K., Daly K.M., O’Regan O., Ryan F.J., Draper L.A., Plevy S.E., Ross R.P. (2019). Whole-virome analysis sheds light on viral dark matter in inflammatory bowel disease. Cell Host Microbe.

[78] Tetz G., Tetz V. (2016). Bacteriophage infections of microbiota can lead to leaky gut in an experimental rodent model. Gut Pathog..

