# Supplementary material for: Differences in Gut Virome Related to Barrett Esophagus and Esophageal Adenocarcinoma
Source: Microorganisms. 2021 Aug 10;9(8):1701. doi: 10.3390/microorganisms9081701 (PMC8401523; doi:10.3390/microorganisms9081701)
Supplement: Supplementary file 1 [file microorganisms-09-01701-s001.zip › Supplematary Figures.pdf]

Supplementary figures

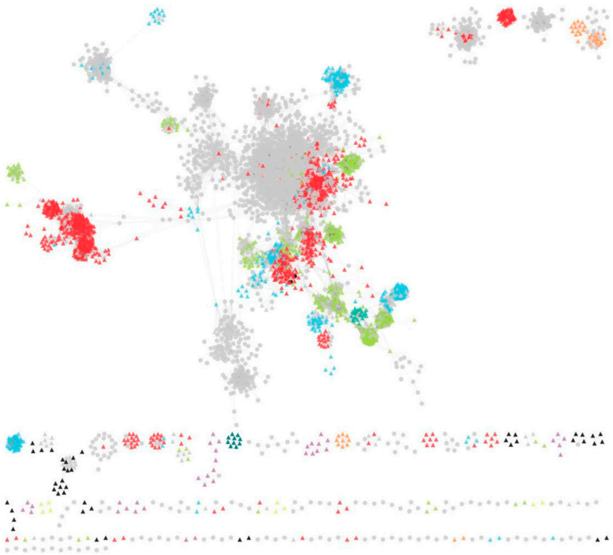

Figure S1. Gene-sharing taxonomic network of viral sequences in this study, including viral RefSeq viruses v85. RefSeq viruses are in triangle shape. Viruses from the *Siphoviridae* family are highlighted in red, *Podoviridae* in cyan, *Myoviridae* in light green, *Microviridae* in orange, *Inoviridae* in purple, *Leviviridae* in yellow, *Fuselloviridae* in dark green, others in black, and unclassified in grey. Each node represents a genome, whereas connecting edges show shared genes between genomes.

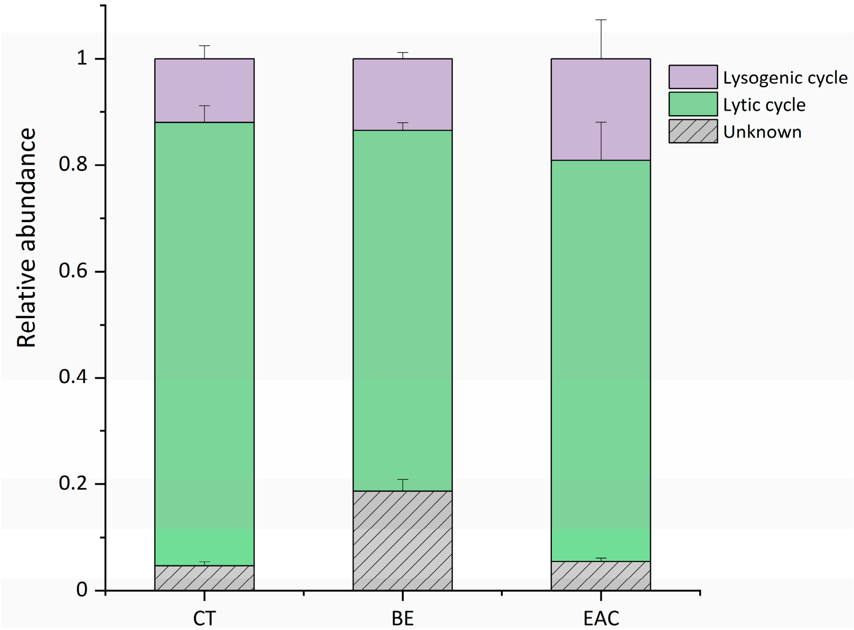

Figure S2. The proportion of lytic/lysogenic replication cycles predicted for the viral contigs from three groups; CT represents stool samples from healthy controls; BE represents stool samples from Barrett Esophagus patients; EAC represents stool samples from Esophageal

Adenocarcinoma patients. Error bars indicate the average±SE. Statistical significance was determined by Kruskal-Wallis, Dunn's post hoc test, $p < 0.05$.

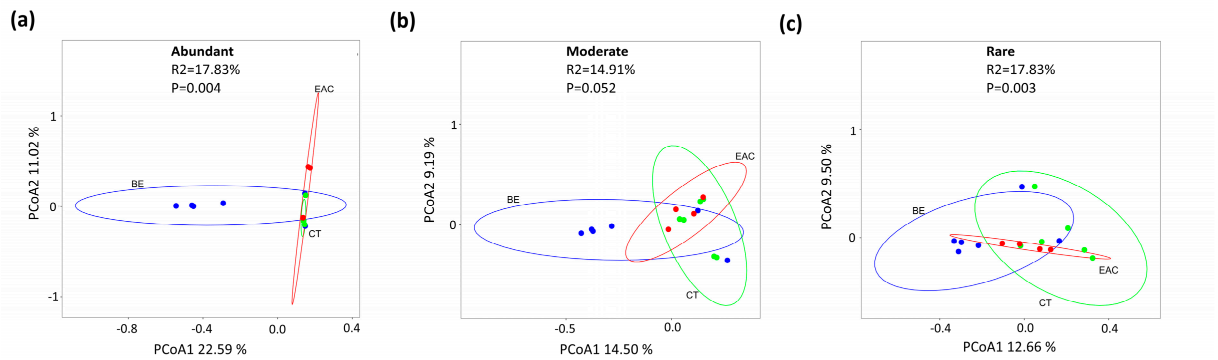

Figure S3. PCoA plot of the viral community composition based on the Bray-Curtis distances in CT, BE, and EAC samples. (**a**) PCoA plot of the abundant viral community composition (**b**) PCoA plot of the moderate viral community composition, and (**c**) PCoA plot of the abundant rare viral community composition. CT represents stool samples from healthy controls; BE represents stool samples from Barrett Esophagus patients; EAC represents stool samples from Esophageal Adenocarcinoma patients. Statistical significance was determined by the PERMANOVA test.

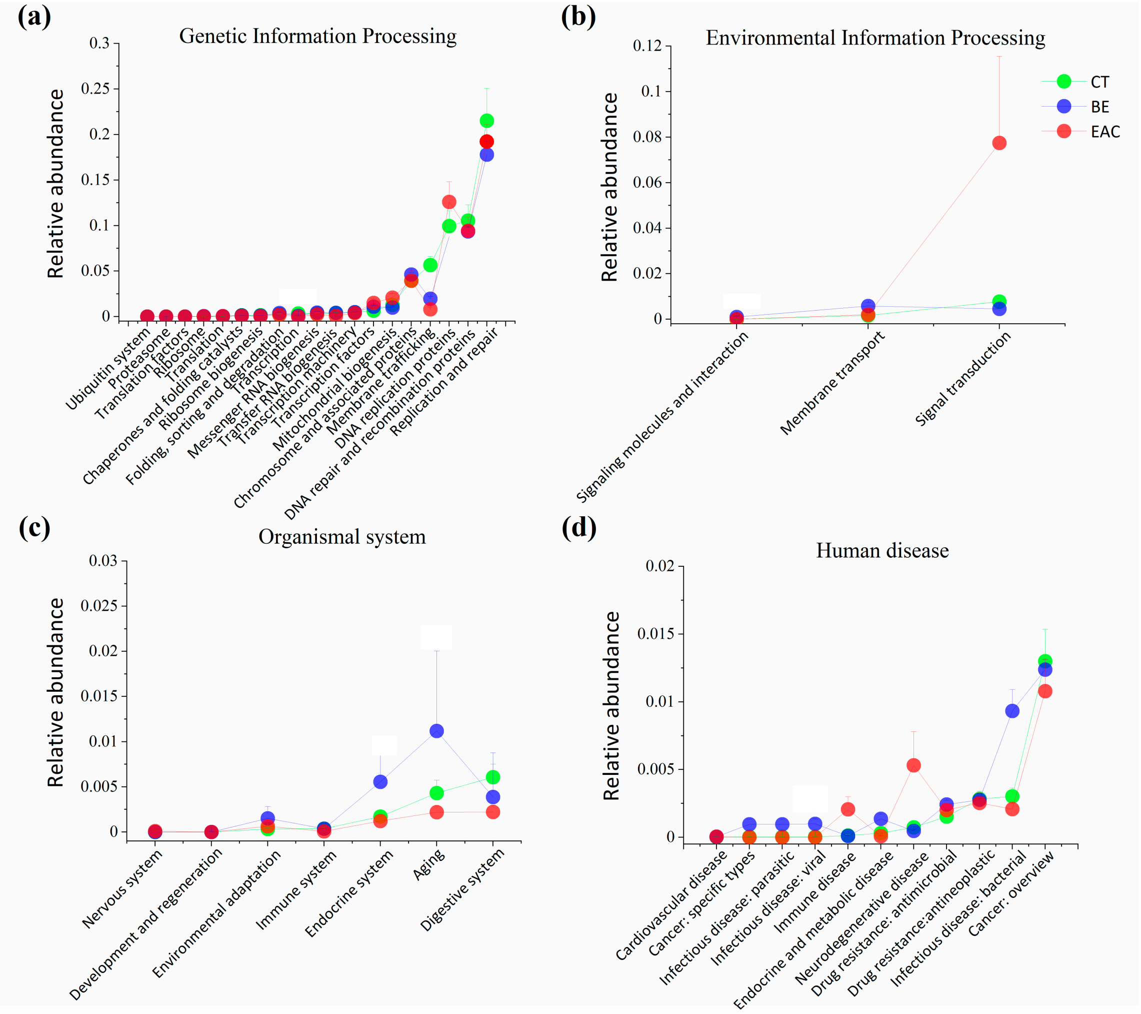

Figure S4. The relative abundance of different functional traits in viral sequences (**a**) The relative abundance of Genetic information processing; (**b**) The relative abundance of Environmental information processing; (**c**) The relative abundance of Organismall system; (**d**) The relative abundance of Human disease. CT represents stool samples from healthy controls; BE represents stool samples from Barrett Esophagus patients; EAC represents stool samples from Esophageal Adenocarcinoma patients. Error bars indicate the average ± SE. Statistical significance was determined by two-way analysis of variance [ANOVA], Tukey's post hoc test, $p < 0.05$.

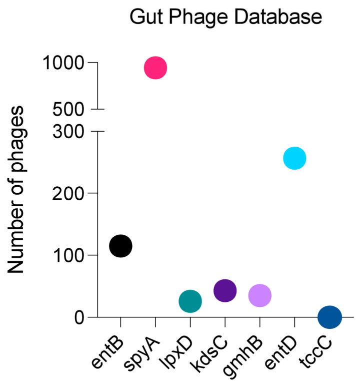

Figure S5. The number of phages that contained the identified AMGs of this study in the Gut Phage Database (GPD).
